# Exploring novel genomic biomarkers for response and survival after neoadjuvant chemotherapy and radical cystectomy of muscle-invasive bladder cancer

**DOI:** 10.1016/j.esmoop.2025.105512

**Published:** 2025-07-14

**Authors:** K. Holmsten, B. De Laere, G. Sjödahl, J. Lindberg, F. Costa Svedman, P. Östling, P. Eriksson, F. Liedberg, A. Ullén

**Affiliations:** 1 Department of Oncology-Pathology, Karolinska Institute, Stockholm, Sweden; 2 Department of Oncology, Capio S:t Görans Hospital, Stockholm, Sweden; 3 Department of Human Structure and Repair, Ghent University, Ghent, Belgium; 4 Department of Medical Epidemiology and Biostatistics, Karolinska Institute, Stockholm, Sweden; 5 Department of Translational Medicine, Lund University, Malmö, Sweden; 6 Department of Pelvic Cancer, Genitourinary Oncology and Urology Unit, Karolinska University Hospital, Stockholm, Sweden; 7 Science for Life Laboratory, Department of Oncology-Pathology, Karolinska Institute, Stockholm, Sweden; 8 Department of Urology, Skåne University Hospital, Malmö, Sweden

**Keywords:** muscle-invasive bladder cancer, neoadjuvant chemotherapy, genomic biomarkers, pan cancer panel DNA sequencing, amplification of chromosome 6p22.3, *FGFR3* mutations

## Abstract

**Background:**

Neoadjuvant cisplatin-based combination chemotherapy (NAC) is standard perioperative treatment of patients with muscle-invasive urothelial bladder cancer (MIBC); however, about half of the patients experience recurrence of the disease. Biomarkers for response and survival represent an unmet medical need. We used tumor specimens from transurethral resections of the bladder to explore genomic alterations and their association with response and survival in MIBC patients treated by NAC and radical cystectomy.

**Patients and methods:**

A pan cancer panel with single-nucleotide polymorphism backbone-based sequencing approach with coverage of the most commonly perturbed cancer genes and low-pass whole genome sequencing was applied for genomic characterization of 110 clinical routine patients treated with NAC before radical cystectomy. Pathological response rates, recurrence-free and overall survival were assessed.

**Results:**

Amplifications of genes on chromosome 6p22.3, particularly of the *E2F3* and *SOX4* gene loci, were associated with improved response and survival to NAC. Patients harboring these alterations had a high pathological treatment response rate and all remained recurrence-free during a median follow-up of 5 years. Conversely, patients with *FGFR3* mutations demonstrated impaired response and survival, whereas *CDKN1A* mutations appeared not related to treatment response but may serve as a biomarker for poor prognosis.

**Conclusions:**

We found the panel-based sequencing approach feasible for exploring genomic alterations associated with clinical benefits of NAC and radical cystectomy. Amplifications of genes on chromosome 6p22.3 and *FGFR3* and *CDKN1A* mutations hold promise as biomarkers associated with response and survival to NAC.

## Highlights

• This study adds to the knowledge on genomic biomarkers for response and survival after neoadjuvant chemotherapy in MIBC.
• A pan cancer panel with SNP backbone-based sequencing approach was applied for genomic characterization.
• Amplifications of *E2F3/SOX4*/*CDKAL1* on chromosome 6p22 were associated with increased response and durable clinical benefit.
• *FGFR3* mutations were associated with impaired response and shorter survival.
• *CDKN1A* mutations were not associated with response but might serve as a molecular biomarker for poor prognosis.

## Graphical Abstract

**Figure:**
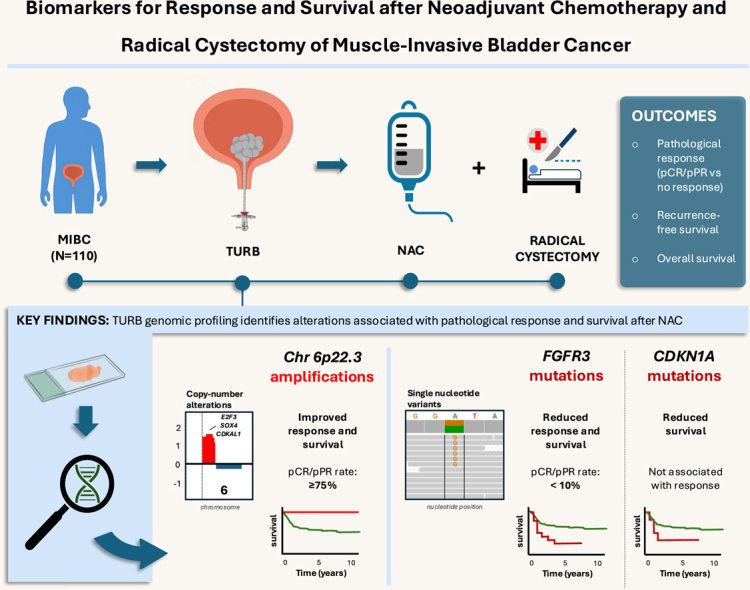

## Introduction

Neoadjuvant cisplatin-based combination chemotherapy (NAC) before cystectomy is an established standard preoperative treatment strategy for patients with muscle-invasive urothelial bladder cancer (MIBC). By use of NAC, a 10% to 16% relative reduction in the risk of death and 5% increase in absolute survival are achieved at 5 years compared with cystectomy alone;^1^, ^2^, ^3^ however, about half of the patients experience recurrence of the disease.

We and others have shown that pathological response is associated with survival in MIBC,^4^^,^^5^ but there is an unmet need to identify novel prognostic and response-predictive biomarkers to further develop precision medicine for this disease. Stratifying patients by their molecular subtype defined by RNA-based classification systems have been explored as one approach to identify biological entities of patients with different clinical benefits from NAC, suggesting luminal-like subtypes (genomically unstable and urothelial-like) to be more responsive.^6^^,^^7^

MIBC is characterized by a high somatic mutation rate^8^ and several studies have investigated the association between clinical outcome following NAC and tumor gene alterations. Specifically, the DNA damage response genes such as *RB1*, *ATM*, *FANCC*, *ERCC2 and BRCA2* and have been explored, but with disparate results.^9^, ^10^, ^11^, ^12^, ^13^, ^14^ While somatic deleterious mutations in *RB1*, *ATM*, *FANCC*,^11^^,^^12^ *ERCC2*^13^ and *BRCA2*^14^ have been associated with outcome following NAC, these genomic alterations and their association with pathological response and survival could not be confirmed in a more recent study with the exception of *ERCC2* and response.^9^ Further, genomic gain-of-function alterations in growth factor receptor genes, in particular *ERBB2* and *FGFR3*, have attracted significant interest in their possible association with clinical benefit of NAC. Mutations of *ERBB2* were associated with response to NAC^15^; however, neither response nor recurrence-free (RFS) or overall survival (OS) could be confirmed in the study by Gil-Jimenez et al.^9^ *FGFR3* alterations are well-characterized in urothelial carcinoma and are of particular interest in view of emerging tyrosine kinase inhibitors targeting the FGFR-receptors. However, studies on the association of *FGFR3* alterations and clinical benefits of NAC have been conflicting regarding both pathological response and survival parameters.^9^^,^^16^ Integrated genomic–transcriptomic–proteomic approaches hold promise for future discovery of novel and refined biomarkers but have not yet been explored in context of NAC for MIBC.^14^^,^^17^

In the present study, we aim to address the unmet need for biomarkers to select MIBC patients who benefit from NAC before cystectomy. A pan cancer panel with a single-nucleotide polymorphism backbone-based sequencing approach with coverage of the most commonly perturbed cancer genes and low-pass whole genome sequencing was applied for genomic characterization. The objectives were to (i) validate in the literature previously suggested predictive and/or prognostic genomic alterations, and (ii) to explore novel co-occurring or mutually exclusive altered genes, and their association to response and survival in MIBC patients treated with NAC and radical cystectomy.

## Patients and methods

### Patient cohort and outcome measures

Patients with MIBC receiving neoadjuvant cisplatin-based combination chemotherapy prior to cystectomy in the Stockholm and Southern Health Care Regions between 2004 and 2014 were included. Clinical data were collected from patient records. Response data were assessed from the pathological report forms after cystectomy. Tumor material for genetic analysis was obtained from the diagnostic transurethral resection of the bladder (TURB) before the onset of NAC. Only patients with complete genomic data were included in the study. The study was approved by the Swedish Ethical Review Authority (Dnr 2013/264 and 2017/37).

The primary dual endpoints were genomic alterations in relation to pathological response in the cystectomy specimen and survival. Genomic alterations were analyzed according to (i) previously suggested predictive and/or prognostic genomic alterations in the literature, and (ii) novel co-occurring or mutually exclusive altered genes. Pathological response was defined as pathological complete response (pCR, i.e. pT0 and pN0) (TNM staging system) or downstaging to partial pathological response (pPR, i.e. pTa, pT1 or pTis and pN0). No response was defined as stable disease or progression at cystectomy (i.e. pT2, pT3, pT4 and/or pN1-3). Recurrence-free survival (RFS) was defined as the time from start of NAC to recurrence or bladder cancer-specific death, whichever came first, and overall survival (OS) as the time from start of NAC to death by all causes.

### DNA extraction, DNA sequencing, and bioinformatics analysis

Formalin-fixed paraffin-embedded (FFPE) sections, from TURB samples obtained before NAC, were used for DNA extraction using the High Pure FFPET RNA Isolation Kit (Roche Molecular Systems Inc., Pleasanton, CA).^18^ Sonication of 15-200 ng tumor DNA was carried out prior to library preparation using Kapa DNA hyper (Roche). Subsequently, in-solution hybridization-based capture, using a pan cancer panel (GMCK v1, Supplementary Table S1, available at https://doi.org/10.1016/j.esmoop.2025.105512), was applied to enable identification of mutations, copy-number alterations and structural rearrangements. Low-pass whole genome sequencing was performed to enable robust copy-number alteration analysis in a subset of FFPE samples that were heavily fragmented. Bioinformatic analysis was performed as previously described.^19^

### Statistical analysis

Descriptive statistics were used to characterize baseline patient and genomic alteration data. Besides assessment and potential recapitulation of the prognostic value of known genomic alterations from literature, novel co-occurring or mutually exclusive target genes were identified by means of pairwise chi-square/Fisher exact testing on genes that were altered in >5% of patients in the investigated cohort. Only significant genes post multiple testing correction by means of Benjamini and Hochberg procedure were used in further analyses. Survival curves for target gene alterations, both RFS and OS were visualized by Kaplan–Meier. Survival differences were determined using the log-rank test. The univariate Cox regression model was used to estimate hazard ratio and 95% confidence interval. The effects of gene alterations on outcome were evaluated in multivariate Cox regression models, *P* values from Wald test, adjusted for the following clinically relevant pre-specified covariates: age, sex, clinical T-stage, and RNA-profiling based molecular subtype (consensus definition^20^). Differences in pathological response [i.e. no response versus partial/complete response (PR/CR)] frequencies between patient populations, based on wild-type (WT) or altered genotypes, were assessed using chi-square or Fisher’s exact tests. All tests were performed in R (version 4.2.2, R Core Development Team, Vienna, Austria), with a two-sided *P*-value <0.05 being considered as statistically significant.

## Results

### Baseline clinical characteristics and treatment efficacy

In total, 110 patients with MIBC were treated with NAC before radical cystectomy. Baseline characteristics and treatment patterns are shown in Table 1. The patients had a clinical T-stage of cT2 to cT4 and no patients had clinical visible lymph node metastases (i.e. all patients were cN0). The patients were treated with cisplatin and gemcitabine (71%) or methotrexate, vinblastine, doxorubicin and cisplatin (38%). The median follow-up was 60.8 months (range 1.5-129.5 months).

**Table 1:** Baseline clinical characteristics, treatment patterns and efficacy

Characteristic	*n* = 110
Age (years), median (range)	67 (37-76)
Sex, *n* (%)	
Male	84 (76)
Clinical stage^a^, *n* (%)	
cT2	45 (41)
cT3	57 (52)
cT4	8 (7)
ECOG performance status, *n* (%)	
0	103 (94)
1	7 (6)
Hb (g/l)^b^, median (range)	138 (93-168)
Consensus molecular subtype (RNA), *n* (%)	
Luminal papillary	30 (27)
Luminal nonspecified	10 (9)
Luminal unstable	19 (17)
Stroma rich	27 (25)
Basal/squamous like	17 (15)
Neuroendocrine like	7 (6)
Lund molecular subtype (RNA), *n* (%)	
Urothelial	45 (41)
Genomically unstable	28 (25)
Basal/squamous like	22 (20)
Mesenchymal like	4 (4)
Small-cell neuroendocrine like	11 (10)
Neoadjuvant chemotherapy regimen, *n* (%)	
MVAC	38 (35)
Gemcitabine/cisplatin	71 (65)
Gemcitabine/carboplatin	1 (1)
Reason to stop neoadjuvant chemotherapy, *n* (%)	
According to plan	97 (88)
Toxicity	6 (5)
Progressive disease	4 (4)
Patient’s wish/other	3 (3)
Pathological pTN-stage, *n* (%)	
pT0N0	33 (30)
pTisN0	5 (5)
pTaN0	1 (1)
pT1N0	6 (5)
pT2N0	20 (18)
pT3N0	13 (12)
pT4N0	11 (10)
pTxN1-3	21 (19)
Pathological response, *n* (%)	
pCR (pT0N0)	33 (30)
pPR (pTis, pTa, pT1 and pN0)	12 (11)
No response (pT2, pT3, pT4 and/or pN1-3)	65 (59)
Recurrence, *n* (%)	46 (42)
3-year RFS, *%*	60
Location recurrence^b^, *n* (%)	
Local recurrence	15 (14)
Lymph nodes	25 (23)
Lung	14 (13)
Liver	14 (13)
Skeletal	14 (13)
Other	10 (9)
Death, *n* (%)	50 (45)
3-Year OS, %	65

ECOG, Eastern Cooperative Oncology Group; Hb, hemoglobin; MVAC, methotrexate, vinorelbine, doxorubicin and cisplatin; OS, overall survival; RFS, recurrence-free survival.

a All patients = cN0.

b One patient missing data.

The clinical treatment efficacy (pathological response, RFS and OS) was comparable with other neoadjuvant cohorts.^1^^,^^21^ The response to NAC was 30% pCR and 11% pPR, respectively, whereas 59% of patients were non-responders. Of the 42% of the patients who recurred, 80% experienced a relapse within 2 years after cystectomy. Approximately half of the patients who recurred received systemic treatment at a later date for metastatic disease. The 3-year RFS was 60% and 3-year OS was 65%, respectively (Table 1). Patients with pCR and pPR had significantly improved RFS and OS compared with patients who had no response to NAC (*P* < 0.001) (Supplementary Figure S1, available at https://doi.org/10.1016/j.esmoop.2025.105512).

### Validation analyses of previously suggested predictive genomic alterations

The genomic alterations detected in the present cohort of MIBC patients treated with NAC prior to cystectomy were observed at prevalence estimates, which are in alignment with previously described genomic landscape studies for urothelial cancer (UC).^8^^,^^22^^,^^23^ The most commonly perturbed genes were *TP53* (57%), *RB1 (33%), KDM6A* (25%), *PIK3CA* (23%), *CDKN2A* (17%), *ARID1A* (16%), *KMT2D* (15%)*, ERBB2* (15%), *ELF3* (12%), *CDKN1A* (11%)*, FGFR3* (10%), *STAG2* (9%), *CCND1* (9%) and *BRCA2* (8%) (Supplementary Figure S2, available at https://doi.org/10.1016/j.esmoop.2025.105512).

Previously suggested genes explored for their potential association with outcome following NAC-treatment in MIBC include *RB1*, *ATM*, *FANCC*, *ERCC2 and ERBB2.*^12^^,^^13^^,^^15^ In our study cohort, similarly to Gil-Jimenez et al.,^9^ we could not validate any association for *RB1*, *ATM*, *FANCC*, *ERCC2 or ERBB2* alterations to pathological response or survival (Supplementary Figure S3, available at https://doi.org/10.1016/j.esmoop.2025.105512).

**Figure 1:**
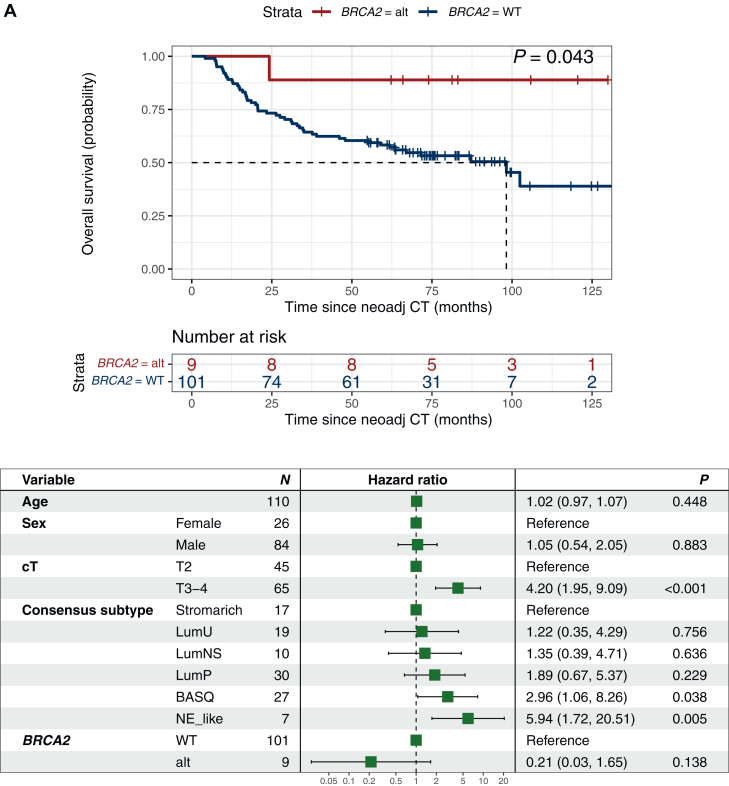

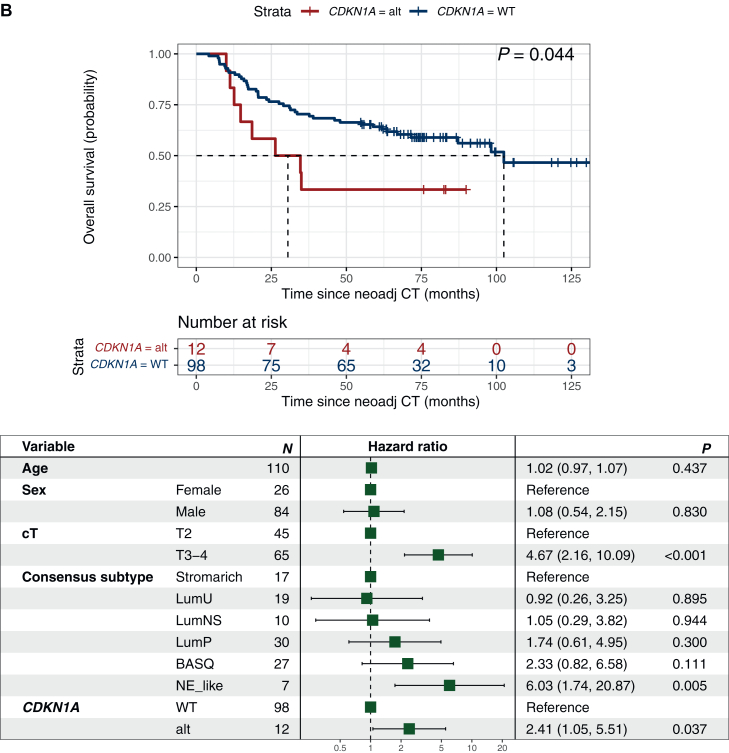
***BRCA2* and *CDKN1A* mutations are associated with survival in patients with muscle-invasive bladder cancer treated with neoadjuvant chemotherapy.** Overall survival for patients with (red) and without (blue) mutations and Forest plots for overall survival adjusted for age, sex, clinical T-stage and RNA-profiling based molecular subtype in (A) *BRCA2* (HR 0.17, 95% CI 0.83-44) and (B) *CDKN1A* (HR 2.15, 95% CI 0.22-0.99). Survival differences by Kaplan–Meier; *P* values from log-rank test. Uni- and multivariate Cox regression analyses for overall survival; *P* values from Wald test. CI, confidence interval; alt, altered; HR, hazard ratio; NeoAdj CT, neoadjuvant chemotherapy; WT, wild-type; cT, clinical T-stage by TNM.

Next, other genes previously suggested to be associated with NAC response or outcome in MIBC were evaluated. *BRCA2*-mutated tumors were reported to be associated with increased response by Taber et al.,^14^ a finding we could not reproduce in the present cohort. However, in line with Taber et al.^14^ we found an association to prolonged OS (*P* = 0.043) in *BRCA2-*altered patients, which lost significance in multivariate analyses when adjusting for baseline age, sex, clinical T-stage, and molecular transcriptomic subtype (Figure 1A, Supplementary Figure S4, available at https://doi.org/10.1016/j.esmoop.2025.105512).

**Figure 2:**
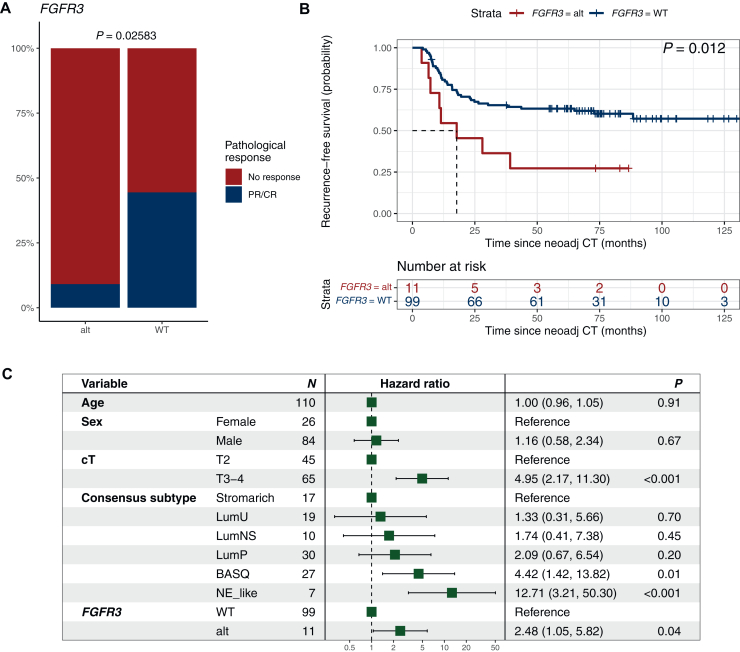

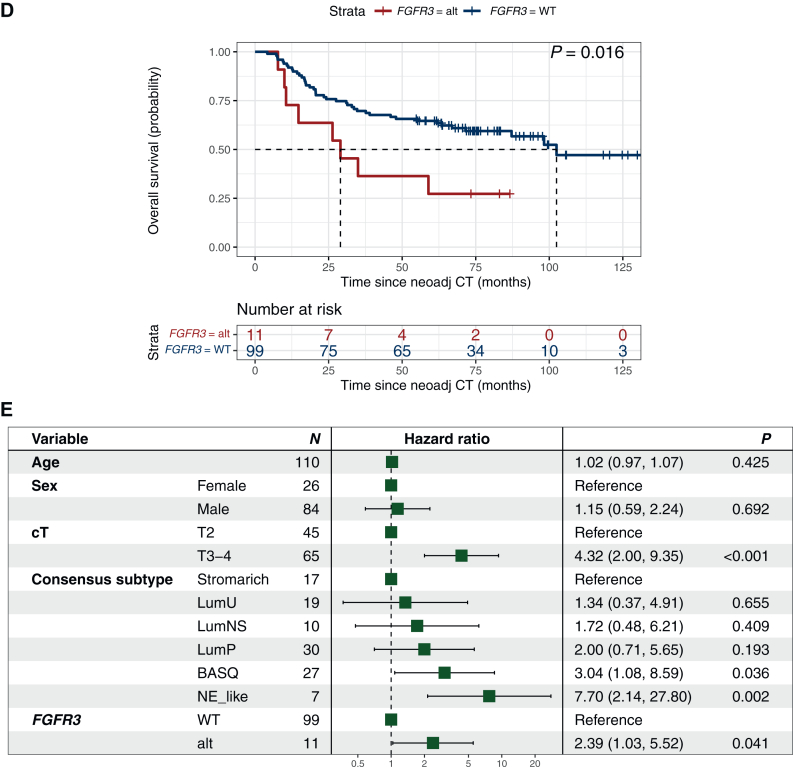
***FGFR3* mutations are associated with decreased response and survival in patients with muscle-invasive bladder cancer treated with neoadjuvant chemotherapy.** (A) Pathological response (PR/CR = pT0 and <pT2) and *FGFR3* status, (B) recurrence-free (HR 2.56, 95% CI 0.18-0.84) and (D) overall survival (HR 2.48, 95% CI 0.19-0.87) for patients with (red) and without (blue) mutations in *FGFR3*. Forest plots adjusted for age, sex, clinical T-stage, and RNA-profiling based molecular subtype for (C) recurrence-free and (E) overall survival. Differences in pathological response by chi-square or Fisher’s exact tests. Survival differences by Kaplan–Meier; *P* values from log-rank test. Uni-and multivariate Cox regression analyses for recurrence-free and overall survival; *P* values from Wald test. No response, stable disease or progression at cystectomy (i.e. pT2, pT3, pT4 and/or pN1-3); PR/CR, downstaging to partial pathological response (PR, i.e. pTa, pT1 or pTis and pN0) or complete response (CR, i.e. pT0 and pN0). Alt, altered; CI, confidence interval; HR, hazard ratio; NeoAdj CT, neoadjuvant chemotherapy; WT, wild-type; cT, clinical T-stage by TNM.

Further, *FGFR3* is mutated in ∼15% of MIBC and suggested to be associated with a worse prognosis.^16^ In the present study, findings by Teo et al.^16^ could be repeated with poor pathological response rates and shorter RFS and OS observed in *FGFR3*-mutant patients, which remained significant in multivariate analysis (*P* = 0.041) (Figure 2).

### Comprehensive genomic profiling identifying novel co-occurring or mutually exclusive genomic alterations

**Figure 3:**
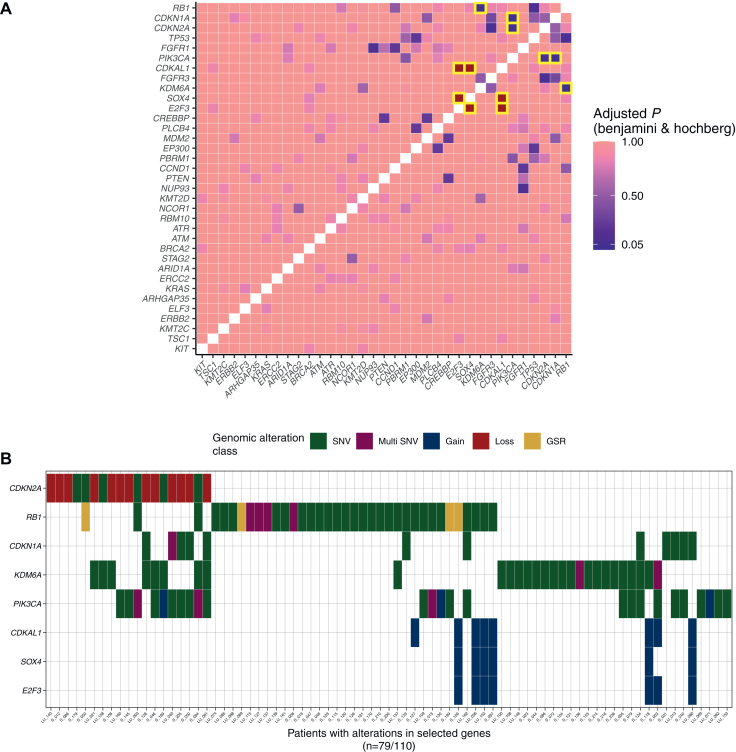
**Generation of a gene target list based on co-occurring or mutually exclusive events.** (A) Co-occurring or mutually exclusive altered genes. Relationships (by means of gradient) with *P* value < 0.05 post Benjamini & Hochberg multiple comparison correction are highlighted with yellow boxes. (B) Oncoprint plot of the gene target list identified in A. In total 79/110 (72%) patients had at least one of these identified genes perturbed. GSR, genomic segmentation rearrangements; SNV, single nucleotide variant.

Next, we identified eight co-occurring or mutually exclusive genomic alterations following pairwise chi-square or Fisher exact testing with multiple comparison correction, occurring in >5% of the analyzed cohort (Figure 3A). These encompassed *CDKN2A* (17%), *RB1* (33%), *CDKN1A* (11%), *KDM6A* (25%), *PIK3CA* (23%), *CDKAL1* (7%), *SOX4* (5%) and *E2F3* (6%) (Figure 3B). Differences in pathological response, RFS and OS were explored for the eight target genes identified. *CDKN1A* and focal amplifications in *E2F3*, *SOX4* and *CDKAL1* on chromosome 6p22.3 showed significant association with response and/or survival whereas the other co-occurring genes did not.

Tumors with *CDKN1A* mutations showed no significant difference in pathological response rate but had shorter RFS (*P* = 0.029) and OS (*P* = 0.044), which remained significant in multivariate analyses (Figure 1B, Supplementary Figure S5, available at https://doi.org/10.1016/j.esmoop.2025.105512).

**Figure 4:**
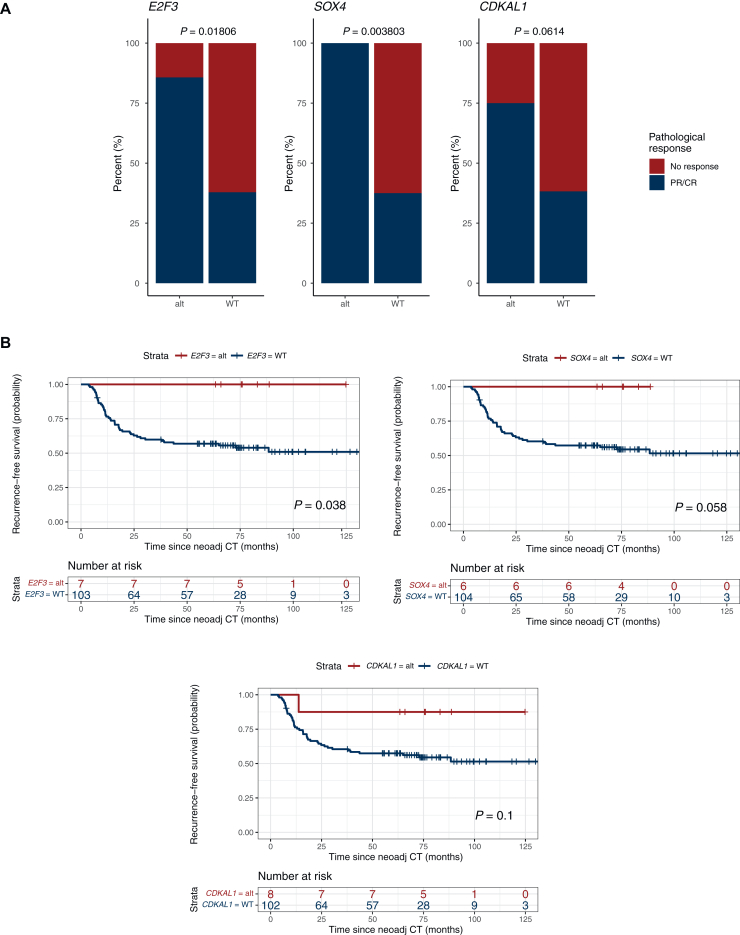

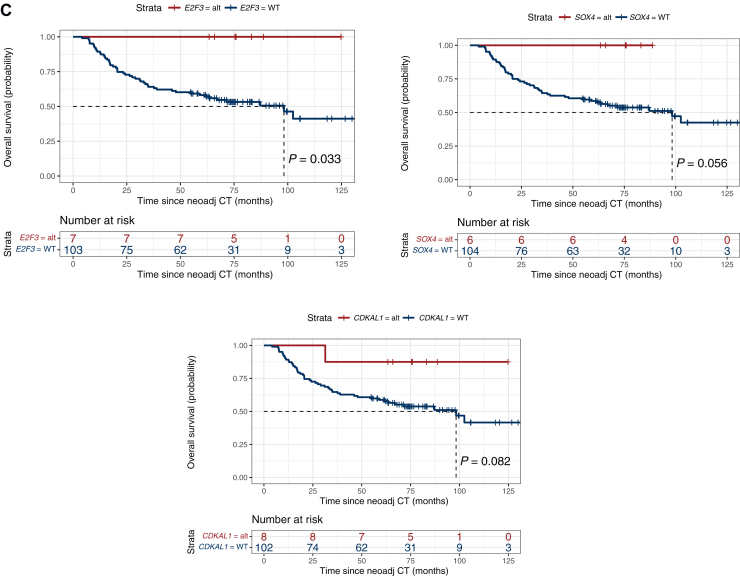
**Focal amplifications of genes on chromosome 6 (*E2F3*, *SOX4* and *CDKAL1*) are associated with increased response and survival in patients with muscle-invasive bladder cancer treated with neoadjuvant chemotherapy.** (A) Pathological response (PR/CR = pT0 and <pT2) and *E2F3*, *SOX4* and *CDKAL1* status, (B) recurrence-free (*E2F3*: HR 1.32 × 10^−8^, 95% CI 0-∞; *SOX4*: HR 1.35 × 10^−8^, 95% CI 0-∞; *CDKAL1*: HR 0.22, 95% CI 0.63-32.91) and (C) overall survival (*E2F3*: HR 1.32 × 10^−8^, 95% CI 0-∞; *SOX4*: HR 1.35 × 10^−8^, 95% CI 0-∞; *CDKAL1*: HR 0.20, 95% CI 0.67-35.36) for patients with (red) and without (blue) focal amplifications in *E2F3*, *SOX4* and *CDKAL1*. Differences in pathological response were determined using chi-square or Fisher’s exact tests. Survival differences by Kaplan–Meier; *P* values from log-rank test. Multivariate analyses were not carried out due to small sample sizes and few events. No response = stable disease or progression at cystectomy (i.e. pT2, pT3, pT4 and/or pN1-3); PR/CR, downstaging to partial pathological response (PR, i.e. pTa, pT1 or pTis and pN0) or complete response (CR, i.e. pT0 and pN0) (pathological stage by TNM). Alt, altered; CI, confidence interval; HR, hazard ratio; NeoAdj CT, neoadjuvant chemotherapy; WT, wild-type.

Interestingly, focal amplifications in *E2F3*, *SOX4* and *CDKAL1* on chromosome 6p22.3, detected in 8/110 (7%) patients, were associated with an improved pathological response rate and increased RFS and OS compared with patients who were copy-number neutral for these loci (Figure 4). These chromosome 6 amplifications were distributed in all the different molecular subtypes by LundTax and the Consensus classification (Supplementary Table S2, available at https://doi.org/10.1016/j.esmoop.2025.105512). The *E2F3* and *SOX4* genes showed a significant association with increased pathological response (*P* = 0.018 and 0.004, respectively), and amplification of *E2F3* was associated with significantly longer RFS (*P* = 0.038) and longer OS (*P* = 0.033). In fact, none of the seven patients with either *E2F3* and/or *SOX4* amplification recurred during a follow-up period of 60.8 months post NAC and radical cystectomy. Given the small sample sizes and limited number of events, no multivariate analyses were undertaken.

## Discussion

In the present study, panel-based sequencing of diagnostic TURB specimens identified focal amplifications of *E2F3*, *SOX4* and *CDKAL1* on chromosome 6p22.3 to be clearly associated with improved clinical outcomes following NAC and cystectomy in MIBC patients. Specifically, significant associations were observed between amplifications of the *E2F3* gene and pathological response rate, RFS and OS. In all, eight patients exhibited these 6p22.3 amplifications, and during follow-up all patients with *E2F3* and/or *SOX4* amplifications remained free of recurrence. Chromosomal 6p22.3 amplifications are well documented in UC, in particular in MIBC, and frequently involve the *E2F3, SOX4* and *CDKAL1* genes.^24^^,^^25^ The *E2F3* gene has previously been suggested to be a key regulator of cell proliferation and the 6p22.3 amplicon a biomarker associated with a more aggressive phenotype in UC.^24^^,^^25^ Furthermore, amplifications of the *E2F3*/S*OX4* genes have been associated with a specific DNA-based clusters in UC and also found in neuroendocrine differentiated tumors.^8^^,^^18^

However, to the best of our knowledge, associations of these 6p22.3 amplifications with clinical outcome following NAC and cystectomy for MIBC have not been previously reported. Given the significant association to pathological response, these 6p22.3 amplifications appear to denote a high proliferative and cisplatin-combination therapy-sensitive genotype, which also resulted in durable RFS and OS outcomes. Notably, the distribution of 6p22.3 amplifications were unrelated to molecular subtypes, indicating a novel subtype-independent genomic treatment predictive biomarker. However, the small sample size and limited number of events in our study precluded multivariate analyses and these observations warrant validation in larger patient cohorts. The applicability of these findings should also be explored in novel systemic regimens recently adopted in the management of advanced UC; immune checkpoint inhibitors (ICI), antibody drug-conjugates (ADC) and ICI/chemotherapy and ICI/ADC combinations, respectively.

Oncogenic gain-of-function *FGFR3* alterations are well described in UC, with reported higher frequencies in low-grade, non-invasive UC tumors and higher frequency in upper urinary tract tumors.^26^^,^^27^ In our homogeneous cohort of MIBC tumors staged cT2-T4N0M0, we found a frequency of *FGFR3* alterations of 11%, which is consistent with previous reports.^8^^,^^9^^,^^16^ The association between *FGFR3* alterations and clinical benefit of NAC and cystectomy has been explored by several other investigators, but with disparate results.^9^^,^^16^ While Gil-Jimenez et al.^9^ did not observe any association with pathological response, RFS or OS, Teo et al. showed *FGFR3* alterations to be associated with impaired pathological response and RFS.^16^ Similarly to Teo et al., we found that only a minority of patients with *FGFR3*-altered tumors had a pathological response to NAC (9%). Further, the poor pathological response translated into poor RFS and OS outcomes, validating previous findings by Teo et al.^16^ and strengthening the hypotheses that *FGFR3*-altered MIBC patients constitute a distinct molecular entity with poor clinical benefit of preoperative cisplatin-based combination chemotherapy. Prospective validation of these observations in larger datasets is called for as well as carrying out randomized trials exploring perioperative treatment with FGFR-inhibitors in patients harboring *FGFR3* alterations, such as the SOGUG-NEOWIN study (EudraCT 2022-002586-15).

Alterations in *RB1, ATM* and *FANCC* and amplifications of *ERBB2* have previously been explored for their potential association with outcome following NAC.^11^^,^^12^^,^^15^ In line with the study by Gil-Jimenez et al.^9^ no significant association for any of these genes with either pathological response or survival were observed in the present study. Further, lack of association between mutations in *ERCC2*^9^^,^^13^ and BRCA2^14^ and survival were confirmed. Overall, the impact of alterations in these DNA damage response genes (*RB1*, *ATM*, *FANCC*, *ERCC2* and *BRCA2*) and the growth factor receptor gene *ERBB2* on the clinical efficacy of NAC remains inconclusive. This may be attributed to inherent differences in the cohort profiles including selection biases for NAC, DNA sequencing methodologies and sample sizes.

Among the other co-occurring or mutually exclusive genes identified in the present study, *CDKN1A* mutations demonstrated a significant association with both shorter RFS and OS, but not with pathological response, implying a potential novel prognostic significance of this gene aberration, which is independent of clinical T-stage^28^ and the RNA-expression subtype.

The present study has some limitations. The relatively small sample size is reflected in wide confidence intervals of the reported hazard ratio point estimates. Additionally, the association between *E2F3, SOX4* and *CDKAL1* amplifications and treatment outcome measures was only assessed in univariate analyses due to the low frequency of these alterations. Furthermore, the retrospective nature of our study design is prone to selection biases, which may have influenced outcome readouts, and the results should therefore be interpreted with caution. The patients included in the present study were from an old cohort, and although the same principles for NAC are applied today, validation in newer and external datasets would strengthen these results. However, to our knowledge no such datasets are available. A strength of our study was the population-based and strictly homogeneous cohort of MIBC with cT2-T4N0M0 tumors, all of which have been selected for treatment with NAC according to the same criteria and guidelines. Further strengths were the long follow-up time, and that the patients were characterized by RNA-expression subtyping using both the LundTax and Consensus classifications systems,^20^^,^^29^ allowing us to benchmark our genomic findings corrected for RNA-expression subtypes in multivariate analyses.

### Conclusion

We found that focal amplification of *E2F3, SOX4* and *CDKAL1* on chromosome 6p22.3 may be a novel biomarker associated with improved pathological responses and survival. Our data suggest that this biomarker may identify MIBC patients with increased probability of achieving pathological response and durable clinical benefit from NAC and cystectomy. In contrast, we provide supportive evidence of impaired pathological responses and worse prognosis of *FGFR3*-altered MIBC patients, warranting the investigation of perioperative treatments other than NAC to be explored in this context. Although *CDKN1A* mutations were not significantly associated with treatment response, these alterations stand out as a possible novel molecular subtype-independent biomarker for poor prognosis. Validation of the present findings is warranted in larger well-controlled prospective datasets. Future studies should also explore the relevance of the present findings in novel perioperative systemic regimens using platinum as backbone in combinations with ICI and ADC therapies.
