# Supplementary material for: Exploring novel genomic biomarkers for response and survival after neoadjuvant chemotherapy and radical cystectomy of muscle-invasive bladder cancer
Source: ESMO Open. 2025 Jul 14;10(8):105512. doi: 10.1016/j.esmoop.2025.105512 (PMC12281969; doi:10.1016/j.esmoop.2025.105512)
Supplement: Supplementary Data [file mmc1.docx]

**A**

**B**

**
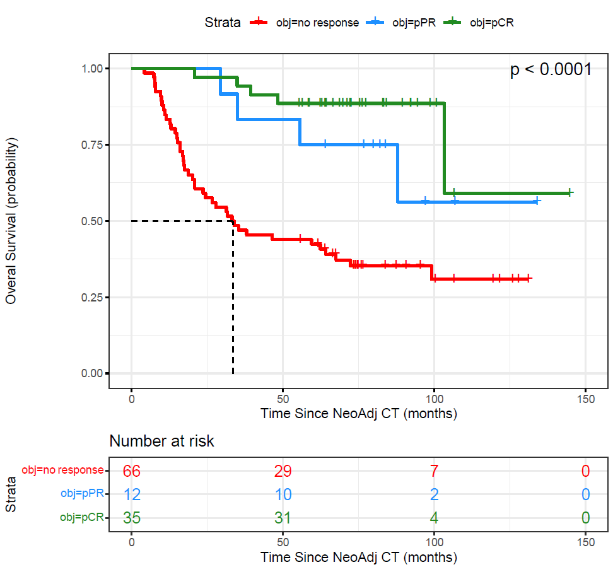

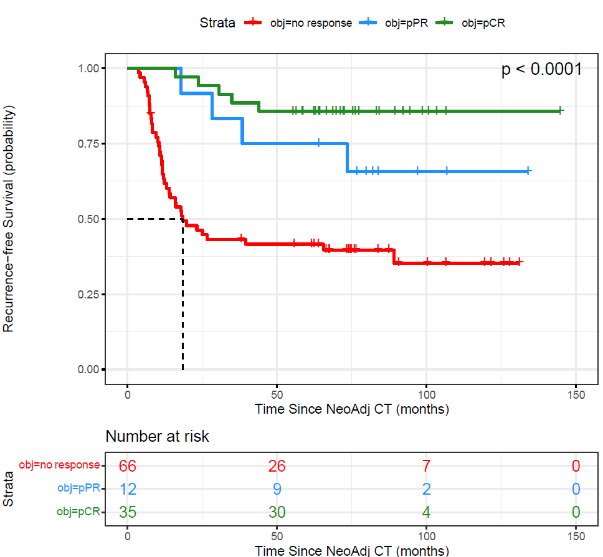
Supplementary Figure S1.** **(A) Recurrence-free survival and (B) overall survival according to pathological response post NAC and radical cystectomy** (i.e. pCR, pPR and pNo-Response). Survival differences by Kaplan-Meier; p values from log-rank test. No response, stable disease or progression at cystectomy (i.e. pT2, pT3, pT4 and/or pN1-3); PR, downstaging to partial pathological response (i.e. pTa, pT1 or pTis and pN0); CR, complete response (i.e. pT0 and pN0); NeoAdj CT, neoadjuvant chemotherapy.


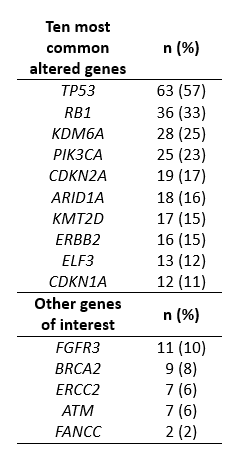


**Supplementary Figure S2. Molecular landscape of the detected genomic alterations in 110 patients with muscle-invasive bladder cancer**. (A) Overview of all detected alterations ranked by observed frequency. (B) Detailed overview of the most commonly (i.e. ≥ 5% of patients) altered genes. SNV, single nucleotide variant; GSR, genomic segmentation rearrangements


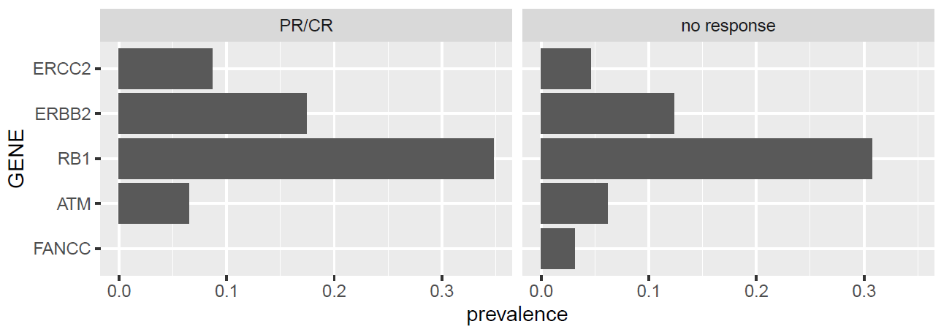

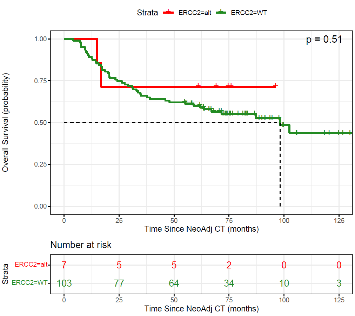

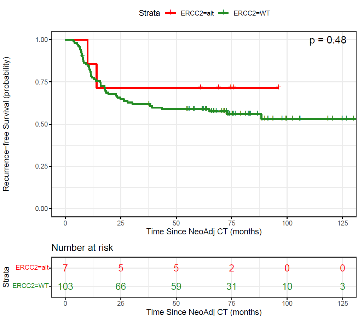

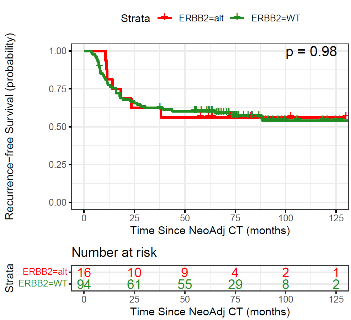

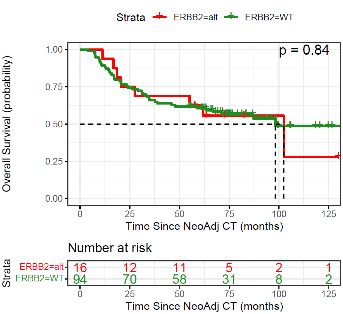

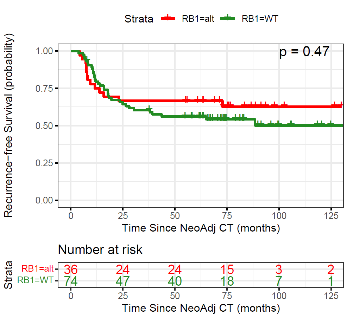

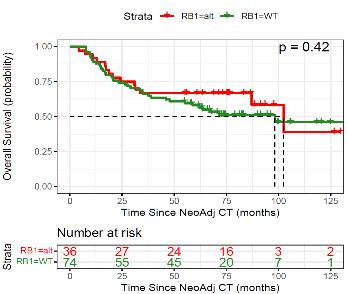

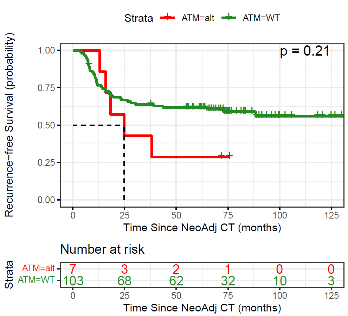

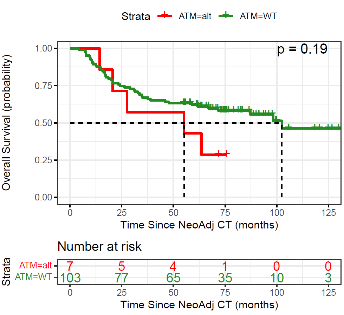

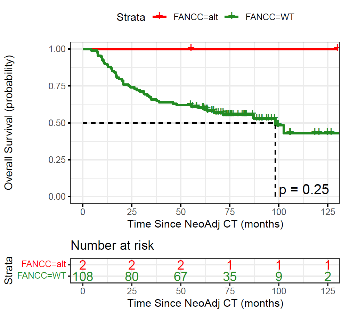

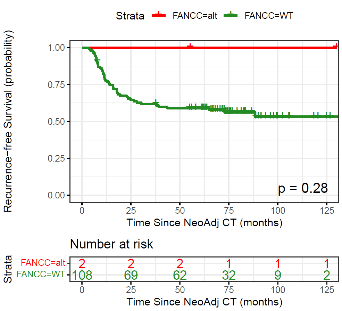


**ERCC2**

**ERBB2**

**RB1**

**ATM**

**FANCC**

**RFS**

**OS**

**A**

**B**

**Supplementary Figure S3.** **(A)** **Pathological response and (B) survival of previously suggested prognostic genes (*ERCC2, ERBB2, RB1, ATM, FANCC*).** Survival differences by Kaplan-Meier; p values from log-rank test. No response, stable disease or progression at cystectomy (i.e. pT2, pT3, pT4 and/or pN1-3); PR/CR, downstaging to partial pathological response (PR, i.e. pTa, pT1 or pTis and pN0) or complete response (CR, i.e. pT0 and pN0); NeoAdj CT, neoadjuvant chemotherapy; alt, altered; WT, wild type; RFS, recurrence-free survival; OS, overall survival.

**A**

**B**

**C**

**D**

**E**


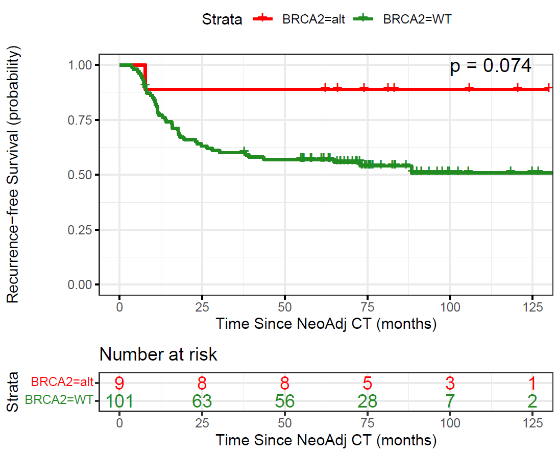

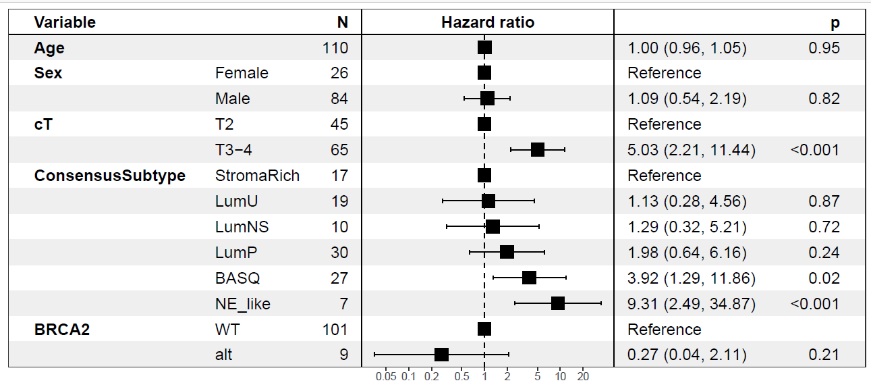

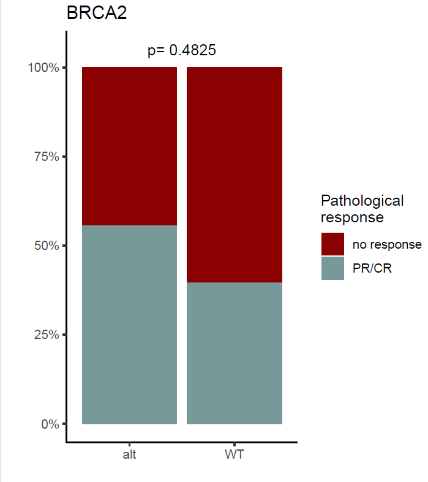

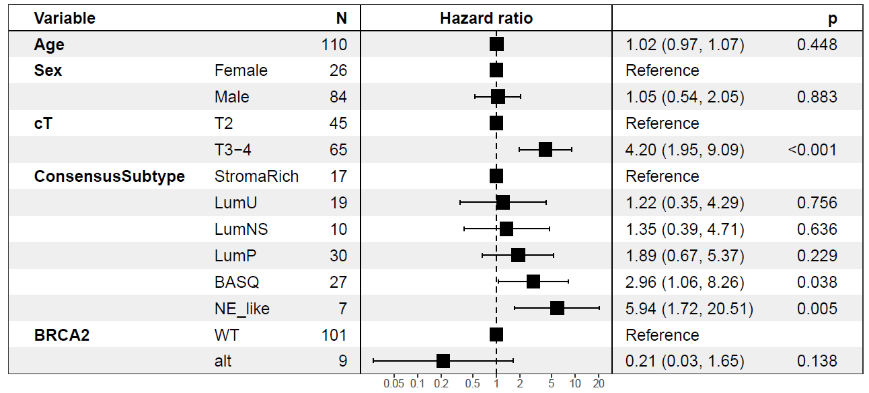

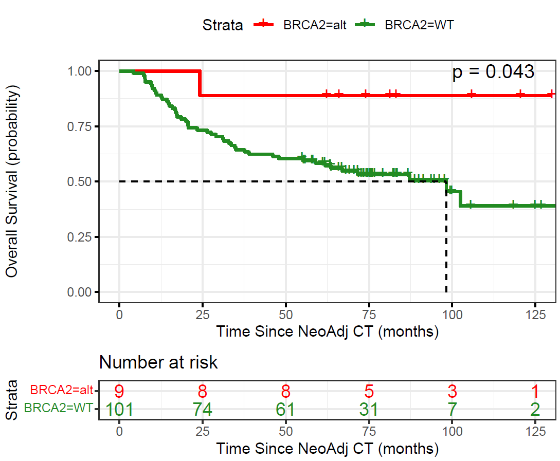


**Supplementary Figure S4. *BRCA2* mutations are associated with increased survival in patients with muscle-invasive bladder cancer treated with neoadjuvant chemotherpy.** (A) Pathological response (PR/CR equals pT0 and <pT2) and *BRCA2* status, (B) recurrence-free (HR 0.20, 95% CI: 0.70-36.83) and (D) overall survival (HR 0.17, 95% CI: 0.83-44) for patients with (red) and without (green) mutations in *BRCA2*. Forest plot adjusted for age, sex, clinical T-stage, and RNA-profiling based molecular subtype for (C) recurrence-free and (E) overall survival. Differences in pathological response by Chi-square or Fisher’s exact tests. Survival differences by Kaplan-Meier; p values from log-rank test. Uni-and multivariate Cox regression analyses for recurrance-free and overall survival; p values from Wald test. No response, stable disease or progression at cystectomy (i.e. pT2, pT3, pT4 and/or pN1-3); PR/CR, downstaging to partial pathological response (PR, i.e. pTa, pT1 or pTis and pN0) or complete response (CR, i.e. pT0 and pN0); NeoAdj CT, neoadjuvant chemotherapy; alt, altered; WT, wild type.

**C**

**B**

**A**

**D**

**E**


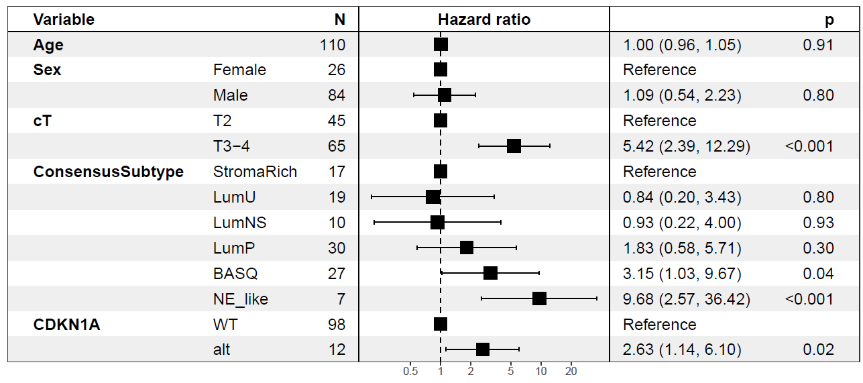

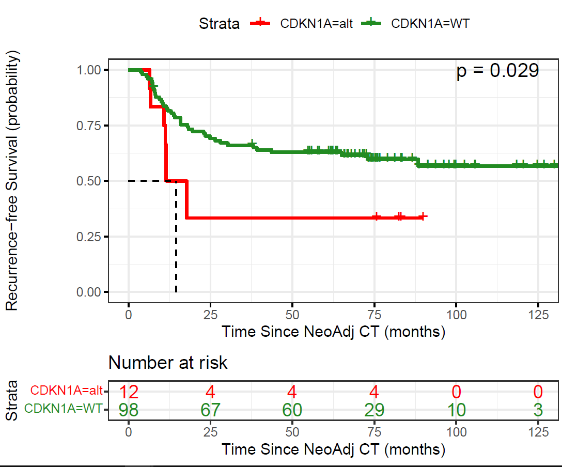

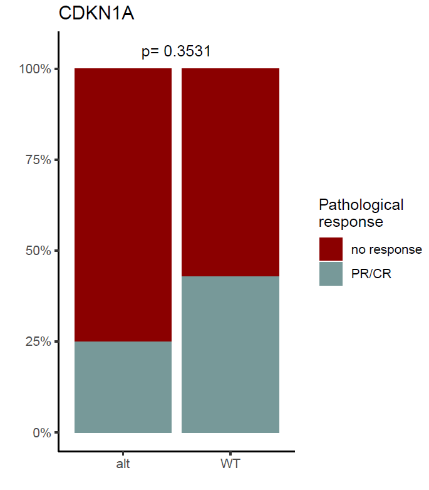


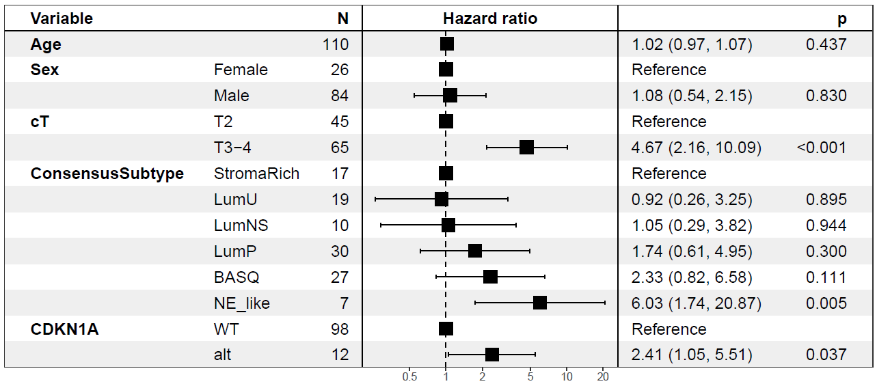

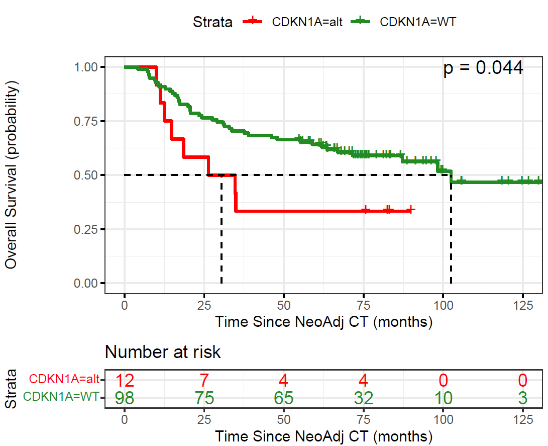


**Supplementary Figure S5. *CDKN1A* mutations are associated with decreased survival in patients with muscle-invasive bladder cancer treated with neoadjuvant chemotherpy.** (A) Pathological response and *CDKN1A* status, (B) recurrence-free (HR 2.29, 95% CI: 0.20-0.94) and (D) overall survival (HR 2.15, 95% CI: 0.22-0.99) for patients with (red) and without (green) mutations in *CDKN1A*. Forest plots adjusted for age, sex, clinical T-stage, and RNA-profiling based molecular subtype for (C) recurrence-free and (E) overall survival. Differences in pathological response by Chi-square or Fisher’s exact tests. Survival differences by Kaplan-Meier; p values from log-rank test. Uni-and multivariate Cox regression analyses for recurrance-free and overall survival; p values from Wald test. No response, stable disease or progression at cystectomy (i.e. pT2, pT3, pT4 and/or pN1-3); PR/CR, downstaging to partial pathological response (PR, i.e. pTa, pT1 or pTis and pN0) or complete response (CR, i.e. pT0 and pN0); NeoAdj CT, neoadjuvant chemotherapy; alt, altered; WT, wild type.

| **Supplementary Table S2. Characteristics of patients with chromosomal 6p22.3 amplifications** | | | | | | | | | | |
| --- | --- | --- | --- | --- | --- | --- | --- | --- | --- | --- |
| **Patient** | ***E2F3*** | ***SOX4*** | ***CDKAL1*** | **LundTax subtype** | **Consensus subtype** | **Histology** | **Clinical stage** | **Pathological stage at cystectomy** | **Recurrence** | **Death** |
| 1 | 0 | 0 | 1 | GU | LumU | Uro | cT3N0M0 | pT4N2 | 1 | 1 |
| 2 | 1 | 1 | 1 | BASQ | BASQ | Uro | cT2N0M0 | pT0N0 | 0 | 0 |
| 3 | 1 | 1 | 1 | GU | LumU | Uro | cT2N0M0 | pT0N0 | 0 | 0 |
| 4 | 1 | 1 | 1 | BASQ | BASQ | Uro | cT2N0M0 | pT1N0 | 0 | 0 |
| 5 | 1 | 1 | 1 | GU | LumU | Uro | cT3N0M0 | pT1N0 | 0 | 0 |
| 6 | 1 | 1 | 1 | UroC | LumU | Uro | cT3N0M0 | pT0N0 | 0 | 0 |
| 7 | 1 | 0 | 1 | ScNE | Stroma rich | Uro + neuroendocrine | cT2N0M0 | pT2bN0 | 0 | 0 |
| 8 | 1 | 1 | 1 | UroC | LumP | Uro | cT2N0M0 | pTisN0 | 0 | 0 |
